# Polyclonal *BRCA2* mutations following carboplatin treatment confer resistance to the PARP inhibitor rucaparib in a patient with mCRPC: a case report

**DOI:** 10.1186/s12885-020-6657-2

**Published:** 2020-03-14

**Authors:** Andrew D. Simmons, Minh Nguyen, Elias Pintus

**Affiliations:** 1 Translational Medicine, Clovis Oncology, Inc., 5500 Flatiron Parkway, Suite 100, Boulder, CO 80301 USA; 2 Berkshire Cancer Centre, Royal Berkshire NHS Foundation Trust, 5 Redlands Rd, Reading, RG15AQ UK; 3 Guy’s Hospital, Great Maze Pond, London, SE19RT UK

**Keywords:** BRCA, Prostate cancer, PARP inhibitor, Poly (ADP-ribose) polymerase

## Abstract

**Background:**

Poly (ADP-ribose) polymerase (PARP) inhibitors are approved for the treatment of breast cancer susceptibility genes 1 and 2 (*BRCA1/2*) mutant ovarian and breast cancers, and are now being evaluated in metastatic castration-resistant prostate cancer (mCRPC). Reversion mutations that restore *BRCA1/2* function have been shown to be responsible for resistance to platinum-based chemotherapy and PARP inhibitors, however there is no information on the sequential use of these agents in prostate cancer.

**Case presentation:**

A patient with mCRPC associated with a germline *BRCA2* mutation was sequentially treated with carboplatin and the PARP inhibitor rucaparib. Genomic profiling of the available baseline tumor and progression blood samples using next-generation sequencing panel tests identified polyclonal *BRCA2* reversion mutations post carboplatin treatment but prior to rucaparib treatment. A total of 12 somatic reversion mutations were detected and ranged from small indels to larger deletions of up to 387 amino acids. These alterations are all predicted to restore the *BRCA2* open reading frame and potentially protein function. The patient received limited benefit while on rucaparib, likely due to these reversion mutations observed prior to treatment.

**Conclusions:**

Here we report a case of a patient with prostate cancer who received a platinum agent and PARP inhibitor sequentially and in whom polyclonal *BRCA2* reversion mutations were identified as the likely mechanism of acquired resistance to carboplatin and primary resistance to PARP inhibition. These findings suggest caution is warranted in sequencing these agents.

## Background

Results from the phase 2 TOPARP study (NCT01682772) suggest that the poly (ADP-ribose) polymerase (PARP) inhibitor olaparib has activity in men with metastatic castration-resistant prostate cancer (mCRPC) who have a deleterious alteration in a DNA damage repair gene, such as *BRCA2* [1]. Recently, preliminary results of the TRITON2 study (NCT02952534) showed that 52 and 44% of evaluable mCRPC patients with a deleterious *BRCA1/2* mutation had a prostate-specific antigen (PSA) response and Response Evaluation Criteria In Solid Tumors response, respectively, when treated with the PARP inhibitor rucaparib [2]. Based on these encouraging results, the U.S. Food and Drug Administration granted Breakthrough Therapy designation to both olaparib and rucaparib in mCRPC, and there are many ongoing studies evaluating these and other PARP inhibitors in patients with prostate cancer.

PARP inhibitors have been approved for the treatment of *BRCA1/2* mutant ovarian and breast cancers. A key mechanism of resistance to PARP inhibitors and platinum-based chemotherapy in these cancers is the acquisition of reversion mutations in *BRCA1/2* that restore protein function [3, 4]. Reversion mutations in *BRCA2* have also been observed in a small number of mCRPC patients treated with PARP inhibitors or carboplatin [5–8]. Acquired reversion mutations in *BRCA1/2* resulting from exposure to platinum chemotherapy are likely to render tumors less sensitive to PARP inhibitor treatment. In a recent study of patients with ovarian cancer treated with rucaparib following platinum, patients without *BRCA1/2* reversion mutations had a significantly longer median progression-free survival than patients with reversion mutations (9.0 vs. 1.8 months; hazard ratio, 0.12; *P* < 0.0001) [3]. However, there are limited data on the combination or sequential use of platinum and PARP inhibitors in prostate cancer.

In this manuscript, we describe a patient with mCRPC and a germline *BRCA2* mutation who was sequentially treated with carboplatin and the PARP inhibitor rucaparib. We profiled the available baseline tumor and progression blood samples using next-generation sequencing panel tests and identified polyclonal *BRCA2* reversion mutations post carboplatin treatment but prior to rucaparib treatment. The patient received limited benefit while on rucaparib, likely due to these reversion mutations observed prior to treatment.

## Case presentation

**Fig. 1:**
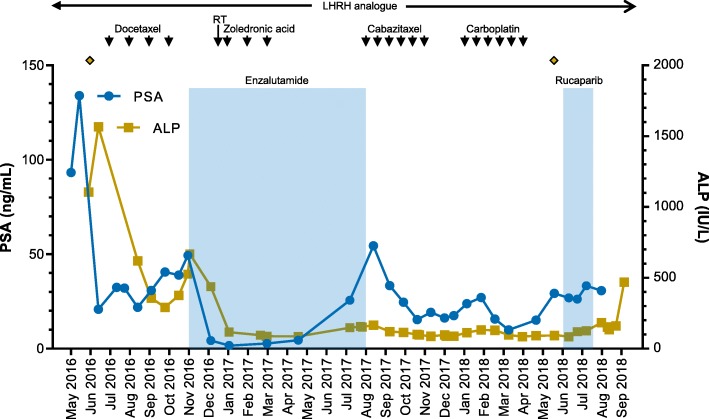
Clinical treatment course and PSA and ALP responses. Treatment and duration of treatment are denoted as arrows or colored areas, and time of sampling as diamonds. ALP, alkaline phosphatase; LHRH, luteinizing hormone-releasing hormone; PSA, prostate-specific antigen; RT, palliative radiotherapy

In May 2016, a 58-year-old patient presented with hematuria and rectal tenesmus. Baseline staging showed prostate cancer invading the mesorectum, pelvic lymphadenopathies, and high-volume bone metastases (T4N1M1); his serum PSA was 136 ng/mL, and his alkaline phosphatase (ALP) was 1106 IU/L (Fig. 1). A prostatic biopsy revealed a Gleason’s 5 + 5 prostate adenocarcinoma. His comorbidities included moderate aortic stenosis, left ventricular hypertrophy, left atrial dilatation, diabetes, hypercholesterolemia, and vitiligo. His Eastern Cooperative Oncology Group (ECOG) Performance Status (PS) was 1.

In June 2016, he commenced on luteinizing hormone-releasing hormone agonists with bicalutamide cover (PSA, 20 ng/mL; ALP, 1567 IU/L) and received his first cycle of docetaxel chemotherapy. In October 2016, docetaxel was discontinued after four cycles due to clinical and biochemical progression. Serum PSA was 41 ng/mL and ALP was 292 IU/L. In November 2016, the patient started on enzalutamide and shortly after received palliative radiotherapy to the lumbosacral spine and started zoledronic acid for prevention of skeletal-related events. He had a marked response to enzalutamide in terms of pain control and PSA and ALP decline (Fig. 1) until August 2017, when due to bone-related pain and PSA and ALP rise, treatment was stopped.

From August to November 2017, the patient received six cycles of second-line cabazitaxel chemotherapy, which were discontinued due to clinical and radiological progression. His ECOG Performance Status for the first time since his diagnosis declined to 2. Based on family history and the aggressive clinical behavior of the disease, in January 2018 he commenced third-line carboplatin chemotherapy (area under the concentration-time curve 5). His initial PSA and ALP levels were 24 ng/mL and 113 IU/L and reached a nadir of 10 ng/mL and 85 IU/L, respectively. Chemotherapy allowed better pain control and improved general condition. He received a total of six cycles of carboplatin, the last given in April 2018. Chemotherapy was discontinued for symptomatic progression and PSA progression, despite a stable ALP level (91 IU/L).

**Fig. 2:**
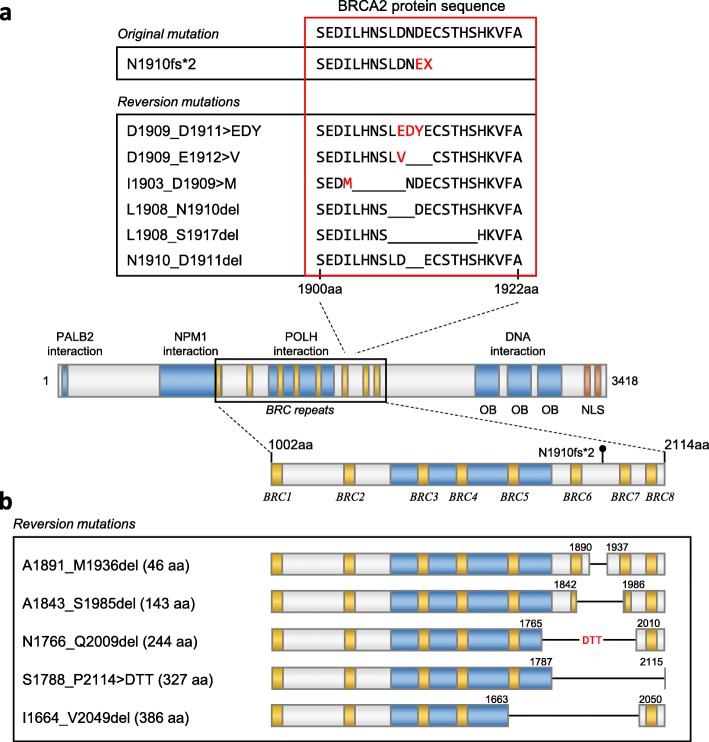
*BRCA2* reversion mutations. Schematic of small indel (**a**) and large deletion mutations (**b**) detected. BRC repeats, interacting regions, and sequences are represented as yellow, blue, and orange boxes, respectively. Substitutions and deletions are represented as red text and black lines, respectively

In May 2018, molecular testing was performed on the prostatic sample taken in June 2016 to determine if the patient was eligible for clinical trials. FoundationONE CDx (version T7) testing [9] identified a deleterious *BRCA2* c.5727_5728insG (N1910fs*2) mutation in the original tumor biopsy (Fig. 2). This alteration was later confirmed to be a germline pathogenic variant in *BRCA2* by Hereditary Cancer Solution testing. Based on published data suggesting that PARP inhibitors are active in patients with *BRCA1/2*-mutant mCRPC [1], in June 2018 the patient commenced on rucaparib 600 mg twice daily (BID) under a compassionate use program due to the lack of an approved standard of care or access to a clinical trial at that time. A baseline plasma sample for circulating tumor DNA (ctDNA) analysis was collected prior to the patient starting rucaparib and profiled using the FoundationACT assay [10]. In addition to the germline *BRCA2* alteration, 12 other *BRCA2* alterations were also observed. Six of the alterations were in close proximity (within ≈10 amino acids) of the original alteration (Fig. 2a, Table 1). All six alterations reestablished the *BRCA2* open reading frame (ORF) by substitutions or short in-frame deletions. Five additional alterations were longer in-frame deletions ranging from 46 to 386 amino acids, four of which resulted in partial or complete loss of the BRC repeat sequences BRC5, BRC6, BRC7, and/or BRC8 (Fig. 2b). These alterations are also predicted to restore the BRCA2 ORF. The final *BRCA2* alteration extended across the exon/intron border: nucleotides 5333–6841 of the coding region and the first 197 nucleotides of the intron (5333_6841 + 197del1706; Table 1). The 1509 base pair deletion within the coding region (6841–5333 + 1) would potentially remove the original mutation as part of an in-frame 503 amino acid deletion.

**Table 1:** *BRCA2* and *CDKN2A* mutations and corresponding variant allele fractions

Gene	Protein	Coding change	Variant allele fraction %
*BRCA2*	N1910fs*2	5727_5728insG	83.7
*BRCA2*	A1843_S1985del	5528_5956del429	0.53
*BRCA2*	A1891_M1936del	5671_5808del138	0.54
*BRCA2*	D1909_D1911 > EDY	5727_5731TAATG > AGACT	0.64
*BRCA2*	D1909_E1912 > V	5726_5735ATAATGATGA > T	0.13
*BRCA2*	I1664_V2049del	4989_6146del1158	0.18
*BRCA2*	I1903_D1909 > M	5709_5727TCTTCATAACTCTCTAGAT > G	0.11
*BRCA2*	L1908_N1910del	5722_5730delCTAGATAAT	0.33
*BRCA2*	L1908_S1917del	5721_5750del30	1.8
*BRCA2*	N1766_Q2009del	5292_6025 > CA	1.3
*BRCA2*	N1910_D1911del	5728_5733delAATGAT	3.3
*BRCA2*	S1788_P2114 > DTT	5362_6340 > GATACCA	1.2
*BRCA2*	Unknown	splice site 5333_6841 + 197del1706	4.8
*CDKN2A*	P114L	341C > T	10.0

**Fig. 3:**
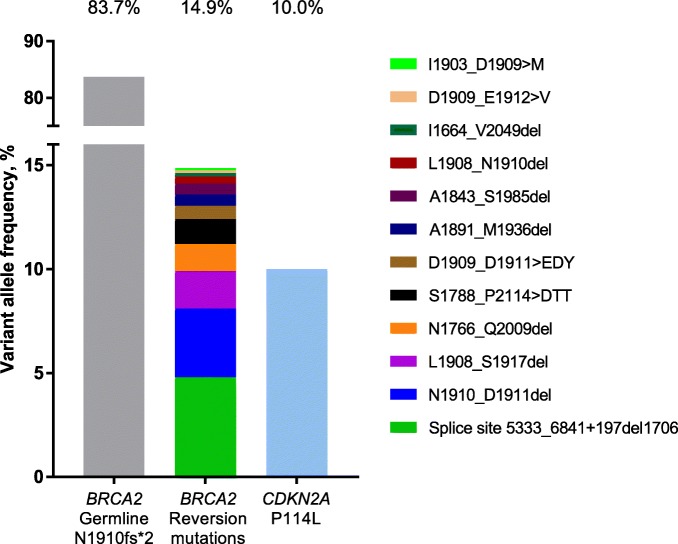
Graph of variant allele fractions for *BRCA2* and *CDKN2A* mutations. *BRCA2* reversion mutations are represented in legend

The FoundationACT assay reported the variant allele fraction (VAF) for the detected alterations (Fig. 3, Table 1). The VAF for the baseline *BRCA2* N1910fs*2 mutation was 83.7%, consistent with the confirmed germline alteration. The VAF of the reversion mutations ranged from 0.11–4.8%, with a total of 14.9%. A CDKN2A P114L alteration was observed at a VAF of 10.0%.

The patient received rucaparib 600 mg BID for a total of 47 days from June to July 2018. His general condition gradually deteriorated. Following hospital admission with sepsis and uncontrolled back pain, imaging confirmed disease progression (new nodal, pulmonary, and hepatic lesions), and rucaparib was discontinued permanently.

## Discussion and conclusions

We report a case of a patient with mCRPC and a germline truncating mutation in *BRCA2* who developed 12 different somatic reversion mutations that restored the protein ORF and would be expected to render the tumor insensitive to platinum-based chemotherapy or PARP inhibitor treatment. Consistent with this hypothesis, the patient had a limited response to subsequent treatment with rucaparib.

Although no definitive conclusions can be made due to the limited sampling, it is likely that the reversion mutations resulted from the 4-month course of carboplatin, as the reversion mutations were not detected in the tumor tissue sample obtained at primary diagnosis. We are also not aware of any reports describing de novo *BRCA2* reversion mutations prior to platinum-based chemotherapy or PARP inhibitor treatment. The emergence of reversion mutations in *BRCA1/2* has been associated with platinum drugs based on their mechanism of action of forming DNA-platinum adducts that leads to DNA lesions [11], whereas it has not been reported in patients treated with taxanes. Although anecdotal, it is remarkable that our patient developed reversion mutations after such a limited exposure to platinum, which suggests a different genomic or biological context in inducing secondary mutations among patients with prostate cancer compared to those with ovarian cancer.

All of the reversion mutations would result in unique, non-wild-type proteins that would restore the C-terminal end of *BRCA2*, including the DNA binding domains, the tower domain, oligonucleotide/oligosaccharide-binding folds, and nuclear localization sequence. However, several of the mutations resulted in large *BRCA2* deletions (up to 387 amino acids), encompassing one or more of the BRC repeats BRC5–8. This region is known to stabilize the RAD51 filament and promote homologous recombination repair upon DNA damage [12]. A previous report suggests that BRC5–8 deletion may confer partial resistance to the DNA damaging agent mitomycin C using *BRCA2*-mutant cell lines [13]. Although the functional consequences of each of the reversion mutations cannot be determined without additional investigation, it is likely that many or all of them restore *BRCA2* function.

The VAF for the *BRCA2* reversion mutations ranged from 0.11 to 4.8%, totaling 14.9% overall. Although the limited activity observed with rucaparib cannot definitively be attributed to these low allele frequency reversion mutations, the identification of polyclonal reversion mutations in prostate cancer patients is consistent with that in other reports [5–8] and highlights the strong selective pressure to restore *BRCA2* function. It is not possible to determine if these alterations are clonal or multiple reversion alterations in a single tumor cell, because shedding may not be similar from each tumor deposit. Interestingly, the patient’s PSA levels remained stable (ranging between 27 and 31 ng/L) throughout rucaparib treatment, indicating that perhaps not all tumor clones contained a reversion mutation and some were responding to treatment.

Another acquired alteration detected in ctDNA following carboplatin treatment was a CDKN2A P114L variant with a VAF of 10%, suggesting that it may have been a somatic tumor-specific variant. The *CDKN2A* gene encodes the p16(INK4A) and p14(ARF) proteins, which both function as tumor suppressors [14]. The P114L (c.341C > T) loss-of-function mutation would prevent p16 from inhibiting CDK4 and inducing cell cycle arrest [15]. *CDKN2A* is commonly altered in patients with metastatic melanoma and cutaneous squamous cell carcinoma [16, 17]. Although *CDKN2A* mutations are rarely observed in prostate cancer, a recent case study reported a *CDKN2A* P81L mutation (which would render the protein functionally defective) as the proposed mechanism underlying acquired resistance to enzalutamide in a patient with CRPC [18]. The emergence of the *CDKN2A* P114L alternation in our patient could have occurred during enzalutamide treatment. However, because the ctDNA sample was obtained after multiple therapies, including carboplatin and cabazitaxel, we cannot rule out the possibility that other agents may have caused the emergence of the *CDKN2A* mutation. Upregulation of cell-cycle pathway observed in patients resistant to enzalutamide along with genomic aberrations in the cell-cycle pathway observed in patients with prostate cancer (such as RB1 loss and CCDN1 amplification) suggest the potential importance of cell-cycle kinases in the development of prostate carcinoma and resistance to enzalutamide [19].

We acknowledge several limitations of our study regarding correlation to patient response. First, the patient’s ECOG PS at the start of rucaparib treatment was 2, and there are limited data on the effectiveness of PARP inhibitors in patients with an ECOG PS > 1. In addition, the patient was exposed to rucaparib for only 6 weeks. Although there is limited information on the time required for mCRPC patients to demonstrate a tumour response to a PARP inhibitor, it has previously been reported that 76% (19/25) of patients with a *BRCA1/2* alteration treated with rucaparib in the TRITON2 trial had a radiographic response within 8 weeks of starting rucaparib [2].

The PARP inhibitor rucaparib is currently being evaluated in patients with mCRPC, where it has shown encouraging antitumor activity. An important question is how to use PARP inhibitors, as well as platinum-based chemotherapies, to maximize the clinical benefit in patients with mCRPC. This case study suggests that caution may be warranted in sequencing these agents.

## Data Availability

All available data generated or analyzed during this study are included in this published article.

## Abbreviations

ALP: Alkaline phosphatase
BID: Twice daily
*BRCA1*: Breast cancer susceptibility gene 1
*BRCA2*: Breast cancer susceptibility gene 2
CDKN2A: Cyclin-dependent kinase inhibitor 2A
ctDNA: Circulating-tumor DNA
ECOG: Eastern Cooperative Oncology Group
LHRH: Luteinizing hormone-releasing hormone
mCRPC: Metastatic castration-resistant prostate cancer
ORF: Open reading frame
PARP: Poly (ADP-ribose) polymerase
PSA: Prostate-specific antigen
RT: Palliative radiotherapy
VAF: Variant allele fraction
